# Isolation of antimicrobial peptides from different plant sources: Does a general extraction method exist?

**DOI:** 10.1186/s13007-020-00687-1

**Published:** 2020-10-23

**Authors:** Anna S. Barashkova, Eugene A. Rogozhin

**Affiliations:** 1 Shemyakin and Ovchinnikov Institute of Bioorganic Chemistry, RAS, ul. Miklukho-Maklaya, 16/10, Moscow, Russia 117997,; 2 Gause Institute of New Antibiotics, ul. Bolshaya Pirogovskaya, 11, Moscow, Russia 119021

**Keywords:** Plants, Antimicrobial peptides, Isolation, Extraction, Liquid chromatography

## Abstract

Plants are good sources of biologically active compounds with antimicrobial activity, including polypeptides. Antimicrobial peptides (AMPs) represent one of the main barriers of plant innate immunity to environmental stress factors and are attracting much research interest. There are some extraction methods for isolation of AMPs from plant organs based on the type of extractant and initial fractionation stages. But most methods are directed to obtain some specific structural types of AMPs and do not allow to understand the molecular diversity of AMP inside a whole plant. In this mini-review, we suggest an optimized scheme of AMP isolation from plants followed by obtaining a set of peptides belonging to various structural families. This approach can be performed for large-scale screening of plants to identify some novel or homologous AMPs for fundamental and applied studies.

## Background

Plants represent a source of biologically active substances with various properties. Some of them can be applied in medicine and agriculture [1, 2]. Antimicrobial peptides (AMPs) are of particular interest among all groups of substances of plant origin. AMPs have several common properties: they are small molecules with a molecular weight of 2–10 kDa that possess amphiphilic properties, and are usually positively charged at neutral and physiological pH values [3, 4]. Meanwhile, AMPs have significant differences in primary and secondary structures. However, it is worth noting that most plant-based AMPs are characterized by the presence of a compact spatial structure, which is achieved by the presence of intramolecular disulfide bonds [3, 5]. This also provides stability in relation to temperature, enzymes, and chemical agents [5]. Plant AMPs are divided into several families based on the similarity of the amino acid sequence, cysteine motifs, and the location of disulfide bonds, as well as secondary structure elements [3, 4]. The main AMP families are defensins, thionins, α-hairpinins (hairpin-like peptides), hevein-like peptides, knottins, snakins, lipid-transfer proteins, and cyclotides. Some peptides do not belong to these families, among them peptides with unusual Cys-motif, lacking disulfide bonds, cyclic peptides without cysteine knot and glycine-, histidine-, alanine-rich peptides [3, 5–11]. According to the Data Repository of Antimicrobial Peptides (DRAMP) (URL: http://dramp.cpu-bioinfor.org/browse/PlantAmpsData.php), currently, more than 800 peptides have been annotated in plants.

AMPs are an important element of the innate immunity of plants, especially to biotic stress factors [5]. AMPs have a wide spectrum of activities (antibacterial, antifungal, insecticidal, and antiviral), and some AMPs also inhibit hydrolases and protein biosynthesis [12]. Due to their chemical properties, plant AMPs also demonstrate antiproliferative action [13, 14]. The above properties of AMPs can be used for the development of new drugs or biological plant protection products [9, 15]. It was established that AMPs are presented in each plant, while each species within a particular taxon (e.g., genus, family) has a certain molecular diversity of peptides belonging to different structural families [16–19]. AMPs can be obtained from all parts of plants: vegetative [20] and generative [19, 21–24], aboveground [17, 25, 26] and underground [27–30]. It has been shown that the largest number and variety of AMP is isolated from seeds [9]. In this regard, seeds and fruits are of the greatest interest as sources of a diversity AMPs.

At present, transcriptomic and proteomic methods in plant AMP research are important and widely introduced [31–33]. Despite this, AMP isolation is still of relevance in investigations of structure–function relationships of AMPs at the cellular and organism levels, when the substance is required as it is [34–36]. Some new peptides that are still not involved in the actual plant AMP classification [4] have been isolated by the classical approach through extraction from plants [7, 28]. So, it seems relevant to summarize all the experience accumulated. The presence of isolation scheme, which allows extracting a wide variety of peptides, as well as comparing the results, seems to give new opportunities in AMP research.

## Plant AMP isolation: general approaches

**Fig. 1:**
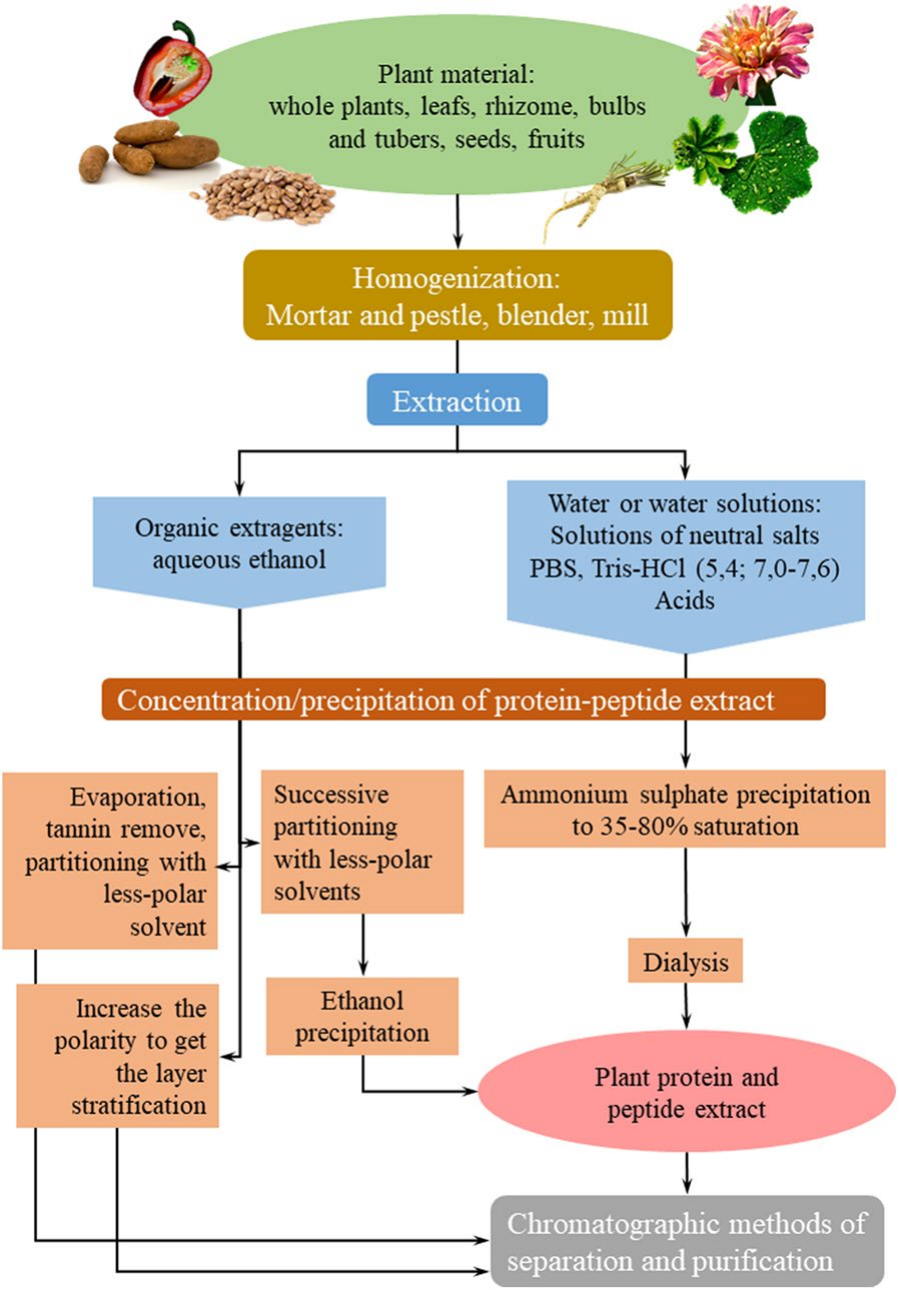
Schematic diagram of AMPs isolation from different plant sources

The first AMP of plant origin was isolated by Okada and Yoshizumi from barley (*Hordeum vulgare*) endosperm in 1970 [37]. Since that time, many approaches to AMP extraction from plant material have been described. There are three main stages of plant AMP isolation: plant material homogenization, extraction, and saturation and purification of the extract. The extract obtained is usually fractionated by a series of liquid chromatography methods (Fig. 1). Each of these stages will be considered in detail.

### Plant material preparation

AMPs can be sourced from different parts of a plant: roots, tubers, bulbs, leaves, flowers, fruits, seeds, or the whole plant. At the stage of homogenization, parts of the plant undergo mechanical destruction. Sometimes plant material is subjected to additional processing before homogenization, if necessary. Vegetative parts of plants are dried or frozen in liquid nitrogen [7, 23, 25, 27, 28], juicy fruits are cleared from seeds [22], seeds are dried, sometimes peeled, and lyophilized [38, 39]. The disintegration method is selected due to the physical properties of the plant material. Seeds, as well as the dried parts of the plants, are ground in a coffee mill. Frozen parts of the plants are crushed in liquid nitrogen using a mortar and pestle. Fruits are ground in a blender in the presence of an extraction buffer. Some plants or their parts accumulate significant content of high and/or low molecular weight metabolites: inulin in roots, storage proteins or fatty oil in seeds, and tannins in leaves and stems. If necessary, additional steps are included in the extraction process, such as heating [40], prefractionating [41], removing excess protein, defatting [42, 43], or removing of tannins [44].

### Choosing of solvent

In the first publications devoted to plant AMP isolation, the general approaches to the protein isolation were implemented. Aqueous solutions of salts, buffer solutions, or diluted acids were used as extraction buffers [45, 46]. Purothionins, the first AMPs obtained from plants, were extracted with a sulphuric acid solution [37]. In some cases, the approach developed and approved for the extraction of peptides of vertebrate origin is applied. Park and colleagues [8] used the previously developed method of AMP isolation from Asian toad (*Bufo bufo gargarizans*) stomach for isolation of glycine- and histidine-reach plant-derived peptides. Later, this method became widely applied in AMP isolation from plants [16, 24, 42, 47, 48].

Currently, two groups of extractants are used to extract AMP from plant sources. The first is represented by water and water-based solutions including salt, acids, and buffer solutions; the second includes organic-based solutions, including water solutions of ethanol (Fig. 1). The most common approaches to AMP extraction are given in Table 1.

**Table 1:** Approaches to the isolation of different structural families AMPs from various plant material

Extractant	AMP	Plant species and part	Extraction method	Reference
Water and water-based solutions				
Water	New Cysteine-Rich Peptides	*Potentilla anserina* (roots)	1. Dried roots were extracted at room temperature for 1 h and centrifuged at 10,000 rpm for 20 min; 2. Concentration by adding ammonium sulfate to 80% relative saturation; 3. Desalting was performed using C_18_ reversed-phase flash column at a stepwise gradient of ethanol (40, 60, 80%)	[24]
	8-Cys hevein-like peptides	Moringa oleifera, (fresh leaves)	1. Plant material was extracted with an equal volume of water while blending (6 min). The mixture was centrifuged at 8000 rpm for 10 min; 2. The supernatant was filtered and loaded on C_18_ flash-column, elution was performed using increase of ethanol concentration (20, 70%)	[41]
Acid solution 50 mM H_2_SO_4_	Defensin	*Nicotiana alata* (flowers)	1. Flowers were ground with liquid nitrogen by mortar and pestle, then extracted with sulfuric acid (3 mL/g wet weight) for 1 h. Insoluble material was filtered and centrifuged (25,000*g*, 15 min, 4 °C) 2. pH of supernatant was adjusted to 7.8 by adding NaOH, and stirred for 1 h, then centrifuged (25,000*g*, 15 min, 4 °C) 3. Concentration by adding solid ammonium sulfate to 80% relative saturation (stirring for 4–16 h at 4 °C) 4. The precipitate was dissolved in gel-filtration buffer, heated to 90 °C and separated using Sephadex G-50 gel-filtration column	[42]
Acid mixture: 1% v/v trifluoracetic acid (TFA), 1 M HCl, 5% v/v formic acid, 1% w/v NaCl	Gly/His-rich peptides	*Capsella bursa*-*pastoris* (roots)	1. Roots were homogenized while blending with extraction mixture (1:4, w:v). Homogenate was filtered through a paper filter and centrifuged at 20,000*g* 30 min 2. The supernatant was concentrated using the reversed-phase C_18_ Sep-Pack cartridge. Peptides were removed from the column by washing with 80% acetonitrile with 0.1% TFA	[6]
Buffer solution: 0.1 M Tris–HCl (pH 7.2)	Defensin-like peptides	*Phaseolus limensis* (seeds)	1. Seeds were washed and soaked in water for 12 h. Then homogenated in a blender with buffer solution. The homogenate was centrifuged at 12,000 rpm for 20 min at 4 °C; 2. The supernatant was fractionated by two-step ammonium sulfate precipitation. In the first step, the solution was saturated to 20%; the resulting supernatant was saturated to 85% 3. After centrifugation at 12,000 rpm for 20 min, the precipitate was collected, dissolved in 100 mL of 0.01 M Tris–HCl buffer (pH 7.2), and dialyzed against the same buffer and subjected to the further separation	[43]
Buffer solution: 10 mM Na_2_HPO_4_, 15 mM NaH_2_PO_4_, 100 mM KCl, 1,5% EDTA, pH 5.4	Thionin-like peptides	*Capsicum anuum* (Fruits without seeds)	1. *C.* *anuum* fruits were extracted with a buffer solution in a 1 to 5 ratio (w:v) for 2 h; 2. The extract was saturated with ammonium sulfate. The precipitate formed between 0 and 70% relative saturation was redissolved in distilled water and heated at 80 °C for 15 min and centrifuged; 3. The resulting suspension was extensively dialyzed against distilled water, freeze-dried and subjected to further fractionation by the chromatographic method	[18]
Organic solutions				
MeOH/CH_2_Cl/0.05% TFA in water (4:4:1)	PawS-derived peptides	*Zinnia haageana* (seeds)	1. Seeds (50 mg) were ground to a fine powder with mortar and pestle under liquid nitrogen with a pinch of 0.1 mm glass beads; 2. The extraction mixture (0.9 mL) was added to the seed powder, and the mixture was vortexed and centrifuged (3 min at 16,000*g*). If the phases were not separated at this point, 0.1 mL of chloroform or 0.1 mL of 0.05% TFA in water was added alternately, followed by short centrifugation after each addition, until phase separation was achieved; 3. After phase separation, the upper polar layer was collected, and dried under vacuum and re-dissolved in 0.5 mL, 5% (v/v) formic acid for the further peptide identification	[44]
EtOH or MeOH 20% or 50% in water respectively	Cyclotydes	*Viola odorata* (aerial parts)	1. Dried plant material was finely ground; 2. Plant material was extracted with an extraction mixture in a 1 to 20 (w:v) ratio for 6 h	[45]
MeOH in water (1:1)	Thionins	*Viscum album* (Green and white parts)	1. Plant material was crushed, the extraction mixture was added (1:5, w:v), the solution was filtered and its volume was reduced; 2. The aqueous phase was successively partitioned with cyclohexane, dichloromethane, and ethyl acetate; 3. Ethanol was added to the concentrated aqueous phase to achieve 85% (v/v) concentration; the precipitate was separated by centrifuging (2000*g*; 10 min); 4. The supernatant was concentrated, and ethanol was added to 85% (v/v). The precipitates were pooled	[46]

#### Water-based solvents

Buffer solutions are the most common extractant for the isolation of plant AMPs. The following principle of AMP extraction and further fractionation is applied. The isolation of the maximum variety of proteins and peptides occurs during the extraction process. Then, separation by mass and charge is used to obtain the peptide fraction. Finally, the characteristics of the individual components are carried out by investigation purposes, if required. Phosphate buffer is the most used extractant for AMP isolation [7, 22, 38, 49–54]. The pH value used is close to neutral (7.4–7.5) or low acidic, close to physiological (5.4–5.5), or lower. When working with seeds with high protein content (cereals, beans, nuts), phosphate buffer saline (PBS) is used with salt concentrations of about 100–200 мM NaCl or KCl [22, 38, 49, 50, 53]. Tris–HCl buffer (10 mM) at pH 7.0–7.6 [17, 29, 41, 55, 56] and acetate buffer at pH 5.0 [30, 57] are also used.

Using the buffer solutions as extractant leads to the extraction of a wide range of polypeptides, as well as other plant metabolites, such as carbohydrates and soluble in water secondary metabolites. This reduces the total yield of the target compounds.

##### Water and aqueous solutions of acids

Most AMPs are cationic molecules [5, 9]. Therefore, during the extraction with aqueous solutions of acids, proteins and peptides with basic properties are extracted first, which simplifies further fractionation. Acidic AMP extraction is the method by which the first thionins from barley (*H. vulgare*) and wheat (*Triticum aestivum*) were isolated. Okada and Yoshizumi used sulfuric acid solution [37]. The first scheme of AMP isolation and purification was proposed simultaneously. Purification presumed a series of reprecipitations. Later, this scheme was modified and applied for defensins isolation [58]. To carry out the acidic extraction, 50 mM H_2_SO_4_ [23, 27, 37, 56, 58] and 2% CH_3_COOH [59] were used. Also, 0.1 M HCl [60] in the presence of 150 мM NaCl can be used to increase the ionic strength of the solution (e.g., when sedimentation of high molecular proteins is required).

Water [28, 39, 61] or neutral salt solution [62] also can serve as solvents for AMPs extraction. The salt solution was used for the first defensin (γ-putothionin) extraction from wheat (*T. aestivum*) endosperm.

Some parts of the plant do not require extraction. When working with coconut water, after the selection of the material, dialysis against the water with acetic acid addition up to pH 2.0 directly follows [63]. However, it must be emphasized that when working with plant material containing a large number of storage proteins, the purification and isolation of the peptide fraction usually requires more stages.

#### Saturation and purification

The next stage after extraction is the saturation and purification of the extract. When using salt buffers, water, or acid solutions as extractants, the saturation of the protein-peptidic fraction is most often carried out by salting out the solution with ammonium sulfate [7, 17, 22, 27, 28, 30, 38, 40, 49–52, 55, 58, 60]. This is carried out in one or two stages [55], if seeds with high total protein content were used. To remove salt excess, dialysis is used. It can be held against water, buffer solution used during the extraction, or solution needed for further analysis. Dialysis is combined with primary purification of low molecular weight impurities using dialysis bags with pores of a certain size (1000–3000 cut-off). In recent years, instead of dialysis, so-called desalination (low-pressure chromatography) is applied. This technique involves the use of hydrophobic sorbents (phenyl-Sepharose, reverse phases C8, C18) [8, 16, 61].

In some cases, the ammonium sulfate saturation is skipped, and the crude extract is fractionated. For example, hevein-like peptides can be isolated by reverse phase chromatography of the crude water extract [39, 61]. Thionins were isolated from white mistletoe (*Viscum album*) by cation exchange chromatography fractionation of the acidic extract after acid neutralization [59]. A lipid-transporting protein from garden pea (*Pisum sativum*) was purified from crude extract after extraction and dialysis [57]. But in most cases, ammonium sulfate saturation is presented. Another method of extract saturation is the addition of trichloroacetic acid and subsequent fractionation using ammonium bicarbonate. This approach was used when the first defensin was isolated [62].

### Organic solvents

Organic solvents are the second group of extractants. The most common variant is the aqueous solution of ethanol. Organic extraction is used if the purpose of the extraction is to isolate a specific group of peptides, especially cyclotides and thionins [26, 64–66]. During the extraction with organic solvents, not only peptides, but also various low molecular weight compounds are extracted to the solution. This should be considered when purifying the resulting extract. The methods of purification, fractionation, and further saturation of the extract can vary (Fig. 1). An extraction scheme of cyclotides was proposed by Claeson et al. [44]. This involved the pretreatment of plant material and additional purification. According to this scheme, plant material was crushed and treated by CH_2_Cl_2_. After treatment, the material was subjected to extraction with aqueous ethanol, and the extract obtained was purified from tannins on a column with polyamide in acidic conditions [25, 44]. Later, this scheme was improved through the optimization of ethanol to water ratio in the extracting solution, the ratio of plant material and extractant, as well as the time and number of repeated extractions [67].

The organic extraction method can be applied to the isolation of other cyclic peptides. In this case, the separation of the peptide fraction is achieved by increasing the polarity of the solution by adding the acid [10]. When using organic solvents for thionins isolation, the aqueous-methanolic extract is subjected to successively partition with solvents of increasing polarity (cyclohexane → dichloromethane → ethyl acetate), followed by precipitation by saturation of the solution with ethanol to 85% [66].

The use of organic solvents for AMP isolation is simple and relatively cheap, and also allows the analysis of small amounts of plant material [10]. However, it is worth noting that when using organic extractants, a limited amount of substances passes into the solution as a whole. At the same time, with a targeted search for AMP substances with certain properties, this method is easier than extraction with aqueous solutions due to the simplification of the fractionation procedure.

The next most common stage of purification of a protein-peptide extract following concentration is the separation by cation exchange chromatography in a linear or stepwise gradient of NaCl.

## Plant AMP isolation: Optimized approach

**Fig. 2:**
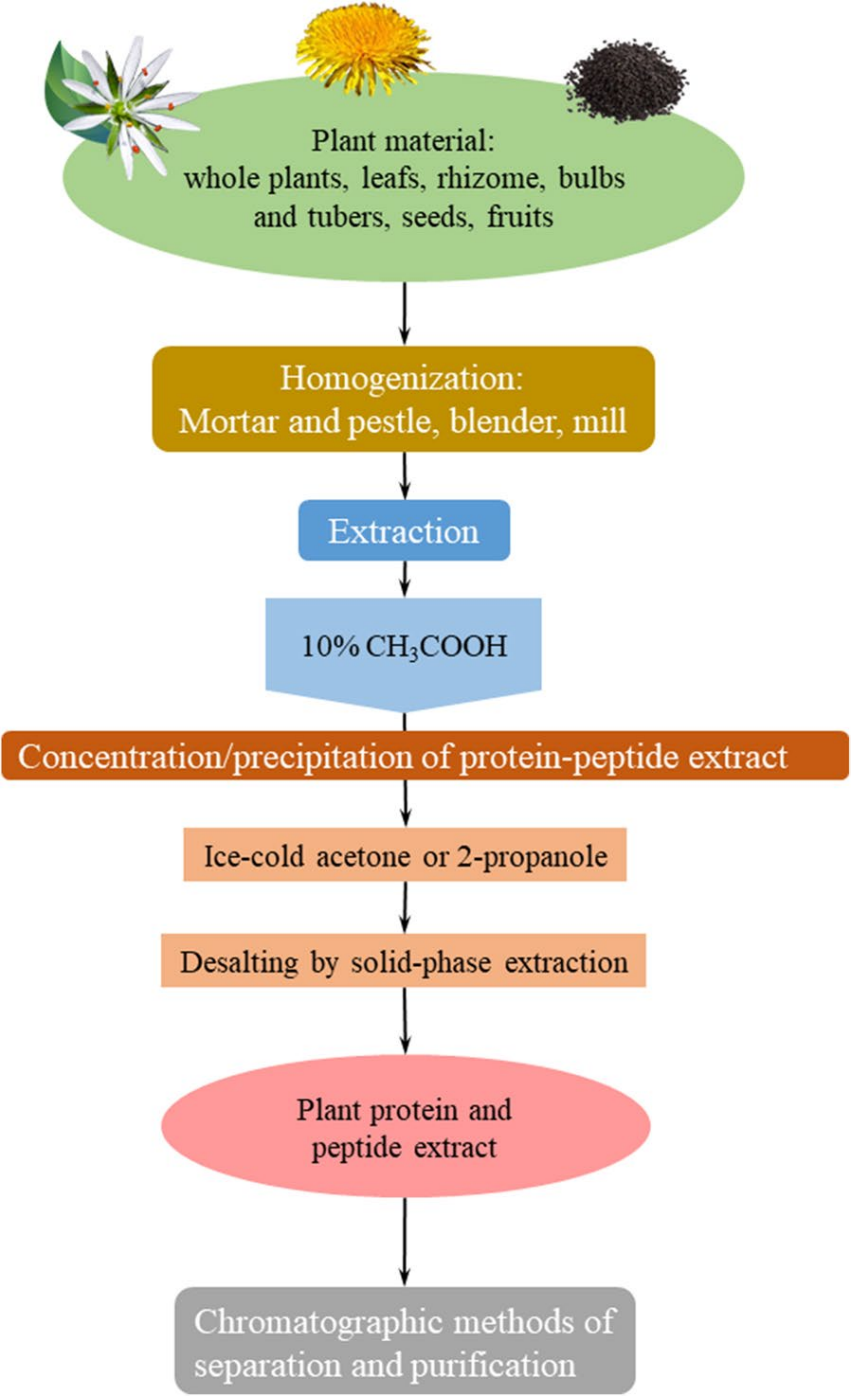
Optimized scheme of plant AMP isolation

An optimized scheme of AMP isolation from plants was proposed in the Laboratory of Neuroreceptors and Neuroregulators of the Institute of Bioorganic Chemistry of the Russian Academy of Sciences (Fig. 2). Acetic acid (10%) is proposed as the extractant. This scheme was used for the extraction of AMPs belonging to different families: α-hairpinins [68–71], defensins [19, 48, 72], thionins [73, 74], and hevein-like peptides [20, 75]. Also, peptides that do not belong to main families were isolated using this scheme, and partially characterized [21, 76, 77].

### Plant preparation and extraction

According to this scheme, plant material is ground in a coffee mill or blender. The extractant used is 10% CH_3_COOH with the addition of a commercial cocktail of proteinase inhibitors (Sigma-Aldrich, USA). This is added to the ground material at a 1:10 (w:v) ratio. Extraction is carried out with constant vigorous stirring for 1 h at room temperature. The mixture is passed through the sieve; fine particles are separated by centrifuging at 4700 rpm for 10 min. The supernatant is filtered through a Whatman paper filter. When large amounts are studied, the resulting supernatant is additionally concentrated approximately 1.5–2-times on a rotary evaporator.

### Precipitation

Organic solvent precipitation is used as a saturation method. Cold acetone (− 70 °C) is poured into the filtrate at a 1:7 ratio while gently stirring; then, the mixture is placed at +4 °C for 6–8 h. After this time, the supernatant is discarded, and if necessary, the suspension is centrifuged at 4700 rpm for 10 min to collect the precipitated fraction. The fraction obtained is dried at room temperature. The dried precipitate was redissolved in 0.1% TFA and purified from low molecular weight components by solid-phase extraction by medium or high-pressure liquid chromatography on a C8 column. The resulting desalted protein-peptide extract is evaporated using a vacuum centrifuge and lyophilized. The obtained lyophilisate can be used in antimicrobial activity tests or subjected to further fractionation. It is worth noting that due to the abandonment of the method of salting out with ammonium sulfate in favour of precipitation with an organic solvent, the described technique allows for the avoidance of dialysis. Thus, the loss of substance can be avoided, and the extraction time can be significantly reduced.

### Fractionation

For further fractionation, the following scheme was proposed. This can be simplified depending on the specific conditions (mainly, the type of biological material used). In the first phase, the fractionation is done by affinity chromatography using heparin-Sepharose sorbent as a solid phase using increasing concentrations of NaCl. This provides the separation of the mixture components by a charge from negatively charged to highly cationic. Solid-phase desalting is carried out. The second step is size exclusion chromatography. This is necessary for plant material with a large amount of total protein, especially for seeds. Sometimes, step two can be avoided if the plant material does not contain reserve proteins. The last step is the fractionating of the mixture by reversed-phase chromatography.

## Conclusion

Antimicrobial peptides can be isolated from plants in various ways, and the experimental conditions can be adapted to the extraction of certain peptides or representatives of peptide families. This approach can be positioned as intensive. However, it eliminates the possibility of screening plants for a wide variety of AMPs, especially potentially undiscovered AMPs. Components of the protein-peptide extract that are not of a special purpose mainly remain unclaimed. The plant AMPs isolation scheme proposed in our group allowed us to obtain and characterize a larger structural diversity of antimicrobial peptides than is represented in most publications on this subject. Nevertheless, some AMPs require alternative isolation approaches (e.g., cyclotides can serve as an example).

Thus, we conclude that there is no universal scheme allowing the isolation of AMPs of all structural families from plants. However, there are two general approaches: cyclic peptides isolation and the isolation of representatives of various AMP families (optimized extraction scheme). Using these algorithms and their optimization can provide a fairly complete diversity of the composition of plant AMPs.

## Data Availability

Not applicable.
